# Supplementary material for: Comparative functional genomic analysis of Alzheimer’s affected and naturally aging brains
Source: PeerJ. 2020 Mar 20;8:e8682. doi: 10.7717/peerj.8682 (PMC7087547; doi:10.7717/peerj.8682)
Supplement: Supplemental Information 1 [file peerj-08-8682-s001.pdf]

Comparative functional genomic analysis of Alzheimer's affected and naturally aging brains

Yi-Shian Peng*[1], Chia-Wei Tang[1], Yi-Yun Peng[1], Hung Chang[1], Chien-Lung Chen[2], Shu-Lin Guo[3,4], Li-Ching Wu[1], Min-Chang Huang[5], Hoong-Chien Lee*[1,5]

[1] Department of Biomedical Sciences and Engineering, National Central University, Taoyuan, Taiwan 32001
[2] Department of Nephrology, Landseed Hospital, Taoyuan, Taiwan 324
[3] Department of Anesthesiology, Cathay General Hospital, Taipei, Taiwan 106
[4] Department of Anesthesiology, Tri-Service General Hospital and National Defense Medical Center, Taipei, Taiwan 114
[5] Department of Physics, Chung Yuan Christian University, Taoyuan, Taiwan 32023

*Co-correspondent author, HCL: hclee12345@gmail.com; YSP: bim962511@gmail.com

Supplementary Figures

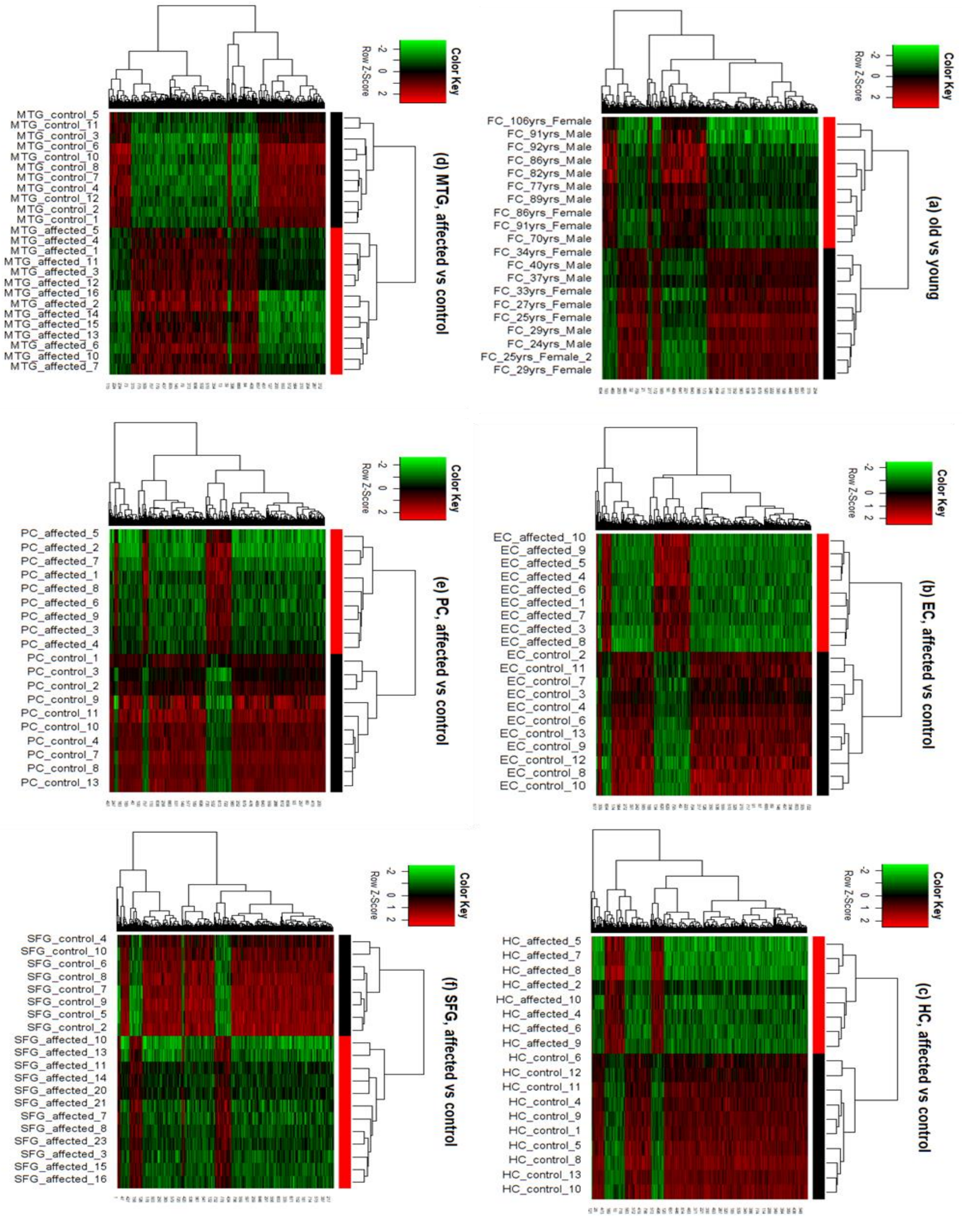

Supplementary Figure 1
Two-way hierarchical clustering of selected datasets For each case the heat map corresponds to a reduced dataset (Supplementary Table 1) obtained by pruning the original dataset so that the PCA score is greater than 0.90 and there is perfect test-control separation in the heatmap. Differentially expressed genes (DEGs) were by LIMMA in R environment with case-dependent FDR: for AG, 7.50E-04; EC, 4.0E-06; HC, 1.0e-05; MTG, 5.0e-06; PC, 1.0e-04; SFG, 1.25e-05. In all cases log2-fold change |FC|>1. (a) AG, or old (70 and older) vs. young (40 and younger). (b) to (f), respectively the five brain regions: EC, entorhinal cortex; HC, hippocampus; MTG, medial temporal gyrus; PC, posterior cingulate; SFG, superior frontal gyrus.

(Supplementary Figure 2, part 1/2)

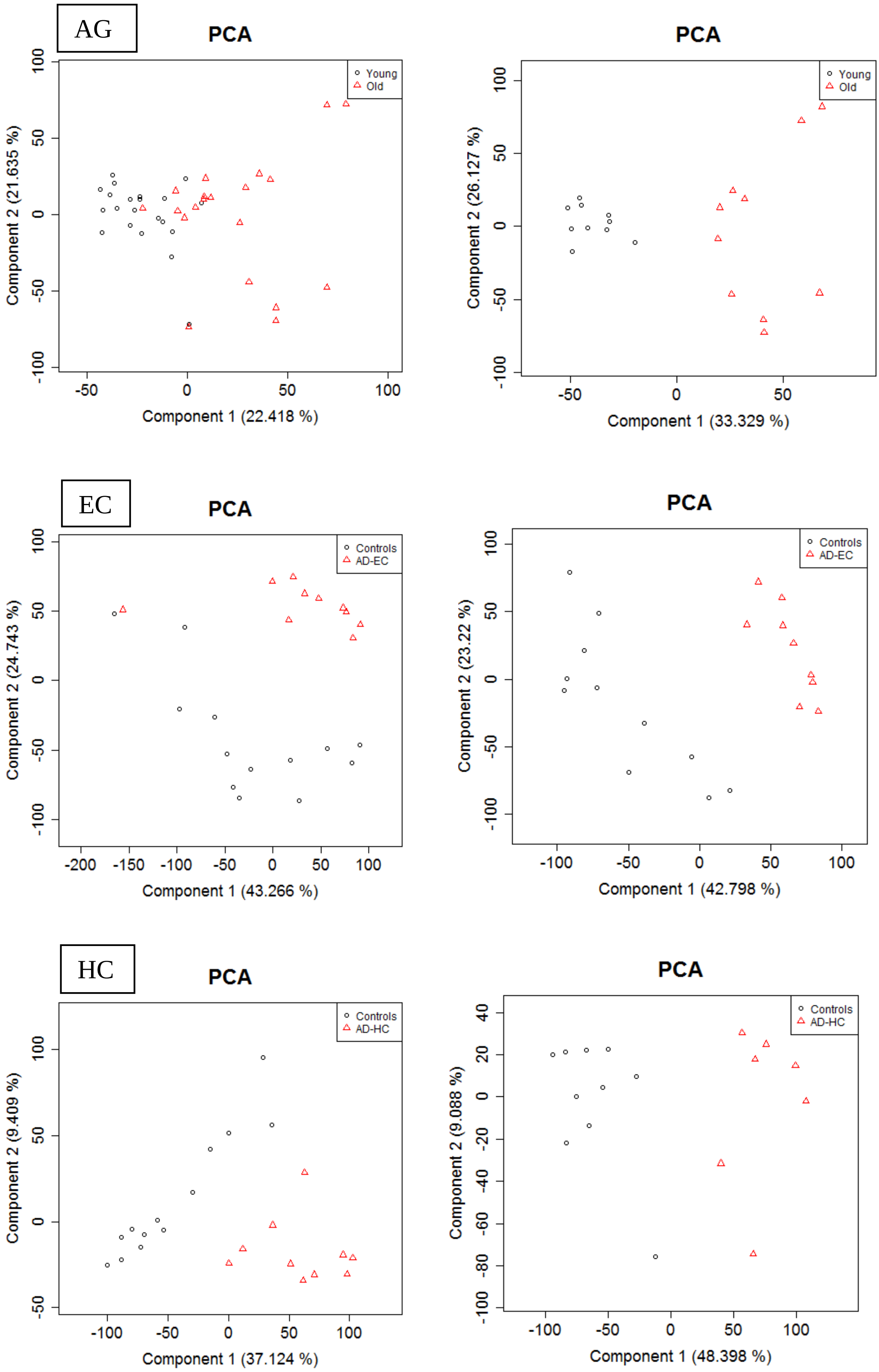

(Supplementary Figure 2, part 2/2)

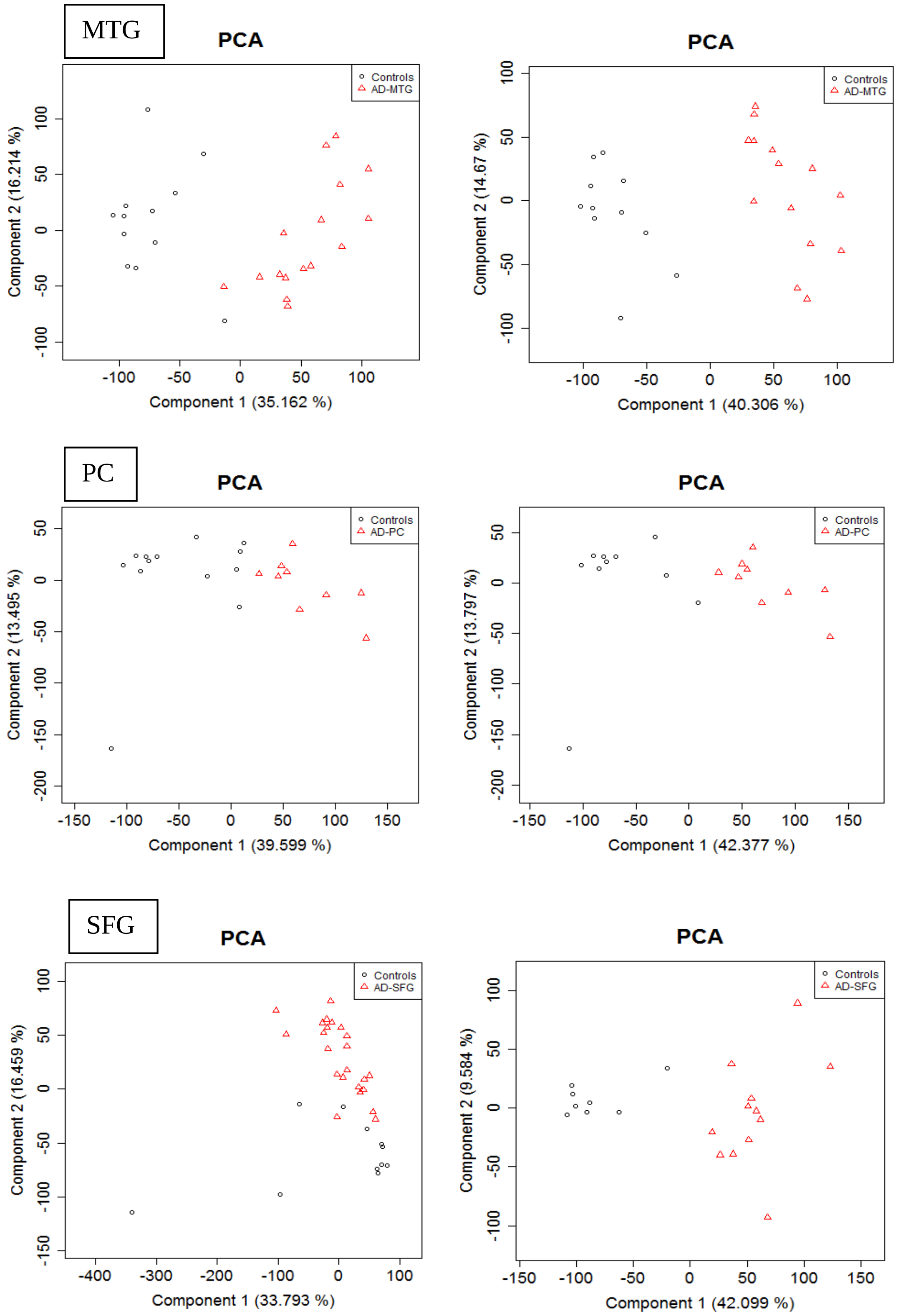

Supplementary Figure 2
PCA plots of the original and reduced datasets For each dataset: left, plot for original set; right, reduced set analyze in this work. Set sizes and PCA scores given in Supplementary Table 1. Abbreviations: AG, normal aging; AD, Alzheimer's disease affected patients; EC, entorhinal cortex; HC, hippocampus; MTG, medial temporal gyrus; PC, posterior cingulate; SFG, superior frontal gyrus.

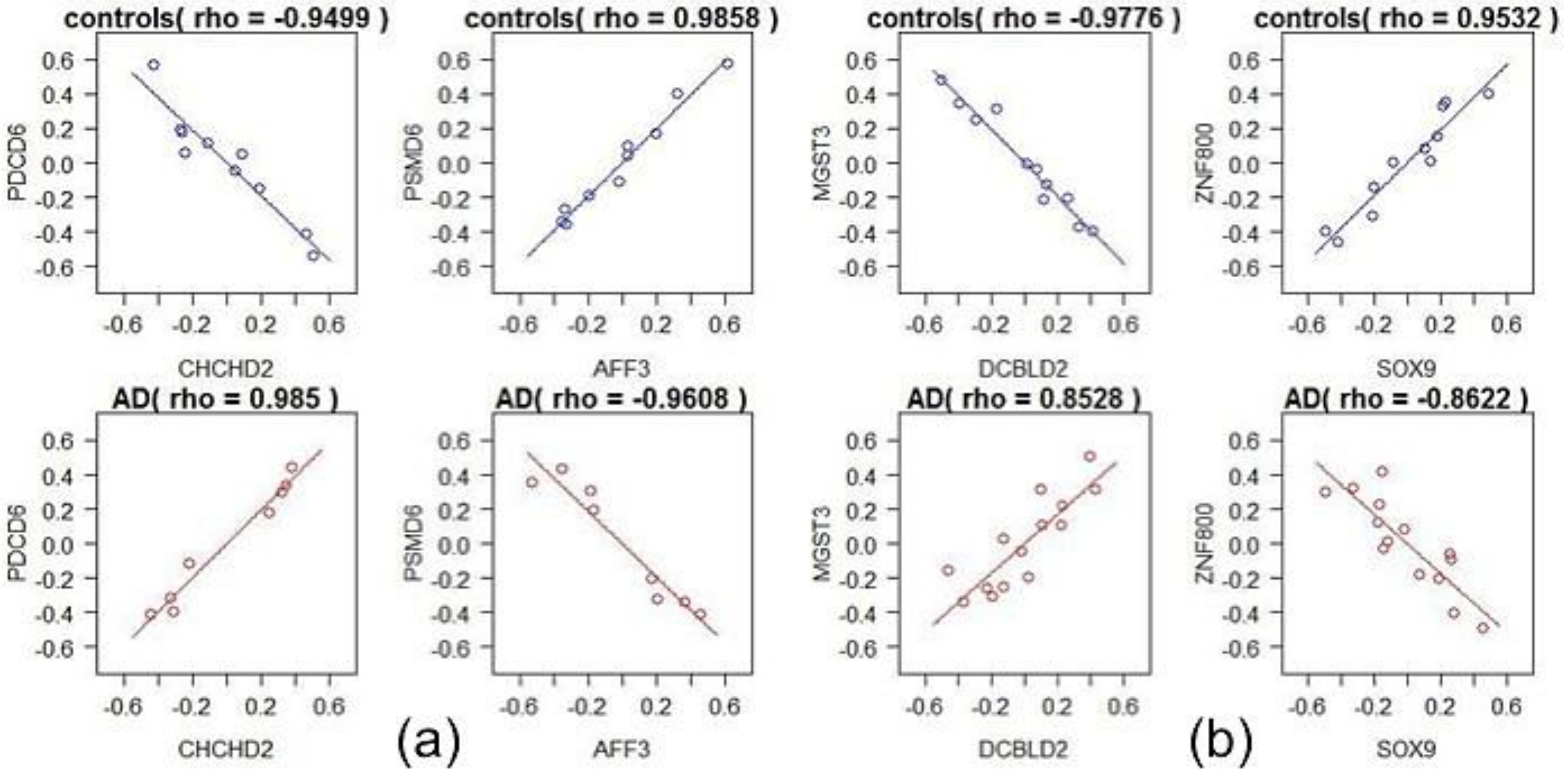

Supplementary Figure 3

Examples of gene-pair correlation plots Plotted along the x- and y-axes are normalized t-statistics of a pair of genes. Each column of two panels gives the plots from control cases (top) and region-specific AD patients (bottom). (a) From the hippocampus (HC) region. The pair CHCHD2-PDCD6 in the left column has negative slope in control and positive slope in HC, hence exhibits GOC. The pair AFF3-PSMD6 in the right column exhibits LOC. (b) From the medial temporal gyrus (MTG) region. The pair DCBLD2-MGST3 (left column) exhibits GOC, the pair SOX9-ZNF600 (right column) exhibits LOC.

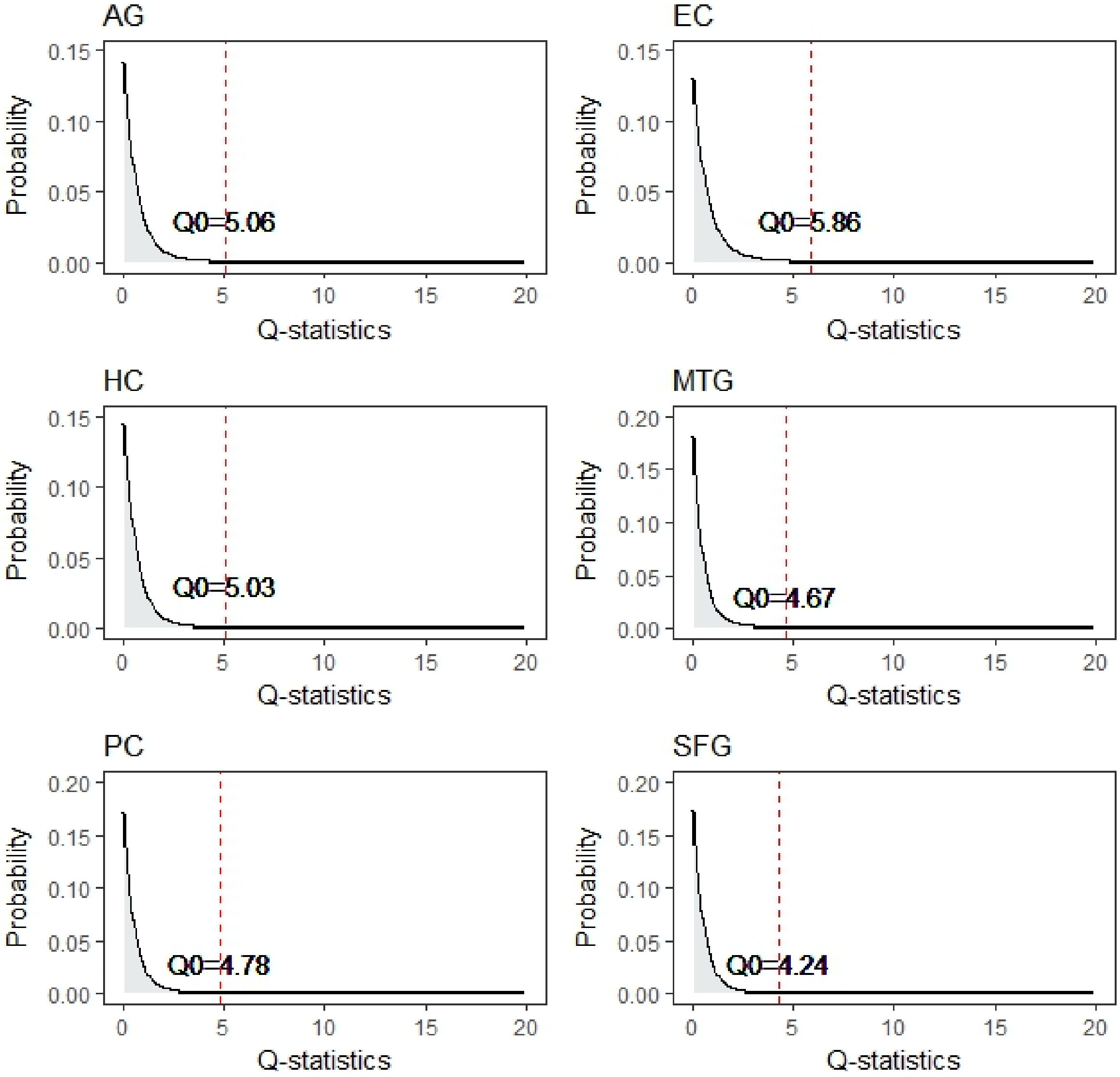

Supplementary Figure 4

Q-statistics distribution of gene-pair correlation in the 6 datasets Pairs with $Q > Q_0$, or those to the right of the red dash line, were selected as DCE gene pairs; they constitute 0.1 % of the total number of pairs. Abbreviations: AG, normal aging; EC, entorhinal cortex; HC, hippocampus; MTG, medial temporal gyrus; PC, posterior cingulate; SFG, superior frontal gyrus.

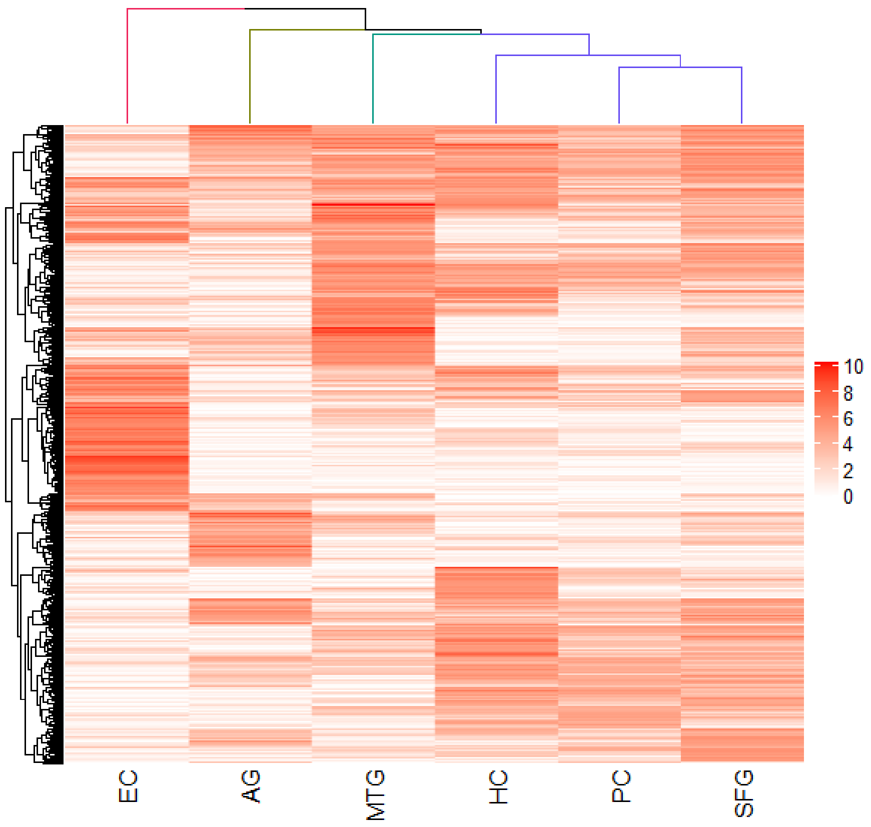

Supplementary Figure 5.

DEG two-way clustering of the six cases The two-way clustering is based on $-\log_{10}(q)$ values of the 3,355 genes in the union of the six curated DEG sets. Abbreviation: AG, normal aging; EC, entorhinal cortex; HC, hippocampus; MTG, medial temporal gyrus; PC, posterior cingulate; SFG, superior frontal gyrus.

Supplementary Tables

Supplementary Table 1. Microarray datasets used in the present study The aging (AG) dataset is old age (>70 y) versus young age (<40 y) and the ALZ datasets are AD-affected patients versus normal from five brain regions: EC, entorhinal cortex; HC, hippocampus; MTG, medial temporal gyrus; PC, posterior cingulate; SFG, superior frontal gyrus. For each case dataset was reduced in size from the original set by pruning arrays to achieve a PCA score of greater than 0.90 and perfect test-control separation in a two-way hierarchical clustering on selected DEGs (by LIMMA, see Supplementary Table 4). Complete samples IDs used in study may be accessed at the Figshare database link: 10.6084/m9.figshare.8952938

Case	Original dataset		Reduced dataset used in study		Reference
	size	PCA score	size	PCA score	
AG	20 vs. 21	0.85	10 vs. 10	1.00	GEO acc. no. GSE53890
EC	10 vs. 13	0.71	9 vs. 11	0.91	GEO acc. no. GSE5281
HC	10 vs. 13	0.88	8 vs. 10	1.00	
MTG	16 vs. 12	0.97	14 vs. 11	1.00	
PC	9 vs. 13	0.91	9 vs. 10	0.95	
SFG	23 vs. 11	0.00	12 vs. 8	1.00	

Supplementary Table 2. Top AD target genes from AlzGene and AlzBase Top "known AD target genes" were selected by frequency of appearance from the databases: from AlzGene top-106 genes with frequency >7, and from AlzBase top-109 genes with >15. Complete lists of the 212 top known AD target genes may be accessed at the Figshare database link: 10.6084/m9.figshare.8952938

Database	Type of genes collection	Gene set used for comparison	Top-5 target genes with highest appearance frequencies
AlzGene (https://www.alzforum.org/)	GWAS	Top-106	*SORL1, APOE, ABCA1, IDE, GAB2*
AlzBase (http://alz.big.ac.cn/alzBase/home)	DEG	Top-109	*NRN1, NCALD, ITPKB, RGS4, GAD1, ATP6V1G2, NSF*

Supplementary Table 3. **Differentially co-expressed (DCE) gene pairs** A DCE gene pair has significant gain or loss in co-expression (GOC or LOC), test relative to control. The Q-statistics cut-off used to select the pairs corresponds to the top 0.1% of pairs in change of co-expression.

Dataset	Total number of DCE pairs	Number of GOC/LOC pairs (% of all)		Q-statistics cut-off
AG	239,220	GOC	151,108 (63.1)	5.06
		LOC	88,222 (36.9)	
EC	238,704	GOC	83,091 (34.8)	5.86
		LOC	155,613 (65.2)	
HC	237,600	GOC	149,576 (62.9)	5.03
		LOC	88,023 (37.1)	
MTG	239,408	GOC	133,800 (55.9)	4.67
		LOC	105,608 (44.1)	
PC	236,919	GOC	63,226 (26.7)	4.78
		LOC	173,693 (73.3)	
SFG	239,346	GOC	124,634 (52.1)	4.24
		LOC	114,712 (47.9)	

Supplementary Table 4. Statistics of curated AG and ALZ gene sets DEGs (differentially expressed genes) were selected by LIMMA in R environment (|FC (log2)| >1 and FDR thresholds: for AG, 7.50E-04; EC, 4.0E-06; HC, 1.0e-05; MTG, 5.0e-06; PC, 1.0e-04; SFG, 1.25e-05); up (down)-regulated DEGs have *t*-score > (<) 0. Gene pairs in an IGN (interacting gene network) had significant gain or loss in co-expression, or GOC or LOC, test relative to control, and known protein-protein interaction. Gene pairs in an xIGN (extended IGN), the same as IGN, but included pairs with interactions once removed, and were required to have minimum degree >2 for AG and >3 for the five ALZ cases. Complete lists of all curated gene sets may be accessed at the Figshare database link: 10.6084/m9.figshare.8952938

Gene sets	AG	EC	HC	MTG	PC	SFG
DEG						
Number of genes	741	845	837	829	764	829
No. of up-regulated genes (number in bracket: % of all)	226 (30)	186 (22)	132 (16)	476 (57)	125 (16)	136 (16)
No. of down-regulated genes	515 (70)	659 (78)	705 (82)	353 (43)	639 (84)	693 (84)
IGN (degree > 0)						
Number of genes	305	325	389	378	335	278
Degree of highest-degree gene	9	11	14	14	6	6
Genes with GOC links	242 (79)	109 (34)	285 (73)	245 (65)	118 (35)	164 (59)
Genes with LOC links	78 (26)	261 (80)	165 (42)	198 (52)	235 (70)	130 (47)
Both GOC & LOC links	15 (5)	45 (14)	61 (16)	65 (17)	18 (5)	16 (6)
xIGN (AG, degree >2; AD, degree > 3)						
Number of genes	901	787	1,063	903	903	664
Degree of highest-degree gene	90	219	270	267	138	221
Genes with GOC links	869 (96)	654 (83)	990 (93)	813 (90)	656 (73)	641 (97)
Genes with LOC links	617 (68)	772 (98)	879 (83)	838 (93)	860 (95)	602 (91)
Both GOC & LOC links	585 (64)	639 (81)	806 (76)	748 (83)	613 (68)	579 (87)

Supplementary Table 5. Top-10 DEGs ranked by false discovery rate (FDR) Up(down)-regulated DEGs have *t*-score >(<) 0. Symbols: #, gene common to two or more brain regions; +, gene is a known AD targets; *, gene is in Alzheimer's disease pathway (KEGG: hsa05010); $, gene in Proteasome (KEGG: hsa03050).

Datasets	DEG	*t*-score	FDR	Datasets	DEG	*t*-score	FDR
AG	RAB3C	-21.8	2.47E-11	MTG	BBX	18.6	8.21E-13
	MYT1L	-20.5	4.09E-11		KLC1	16.2	1.81E-11
	NREP	-16.6	1.61E-09		KTN1	16.1	2.02E-11
	RGS4	-16.5	1.61E-09		FAM120A	15.8	3.13E-11
	LMO4	-15.7	2.94E-09		SEPP1	15.7	3.37E-11
	SYNJ1	-15.5	3.18E-09		RBMS3	14.6	1.61E-10
	PRKCB	-15.0	5.13E-09		LIFR	14.4	1.93E-10
	SERTM1	-14.9	5.44E-09		MSI2	14.4	1.93E-10
	ARPP21	-14.8	5.76E-09		STAG2	14.2	2.48E-10
	FAM126B	-14.6	5.76E-09		ACTB	-13.8	4.27E-10
EC	MRPL41	-23.2	6.13E-12	PC	*UQCRC2	-14.4	4.37E-07
	TECR	-17.5	6.40E-10		*+ATP5B	-12.5	8.80E-07
	SOBP	-17.7	6.40E-10		#GLRB	-12.8	8.80E-07
	ALKBH6	-17.3	6.50E-10		VDAC3	-12.5	8.80E-07
	CDK19	16.6	9.91E-10		#CDC42	-12.5	8.80E-07
	RUNDC3A	-16.3	1.15E-09		+RPL15	-13.1	8.80E-07
	FLJ22184	-15.9	1.50E-09		$PSMA5	-12.1	1.02E-06
	MIB2	-15.9	1.50E-09		MDN1	-12.1	1.02E-06
	BRSK2	-15.5	1.93E-09		SMYD3	-12.1	1.02E-06
	MED25	-15.3	1.96E-09		SRP54	-11.9	1.20E-06
HC	ZNF532	-21.9	4.29E-11	SFG	IQCA1	15.8	2.40E-08
	RTN3	-19.0	4.63E-10		+SNAP25	-15.1	2.99E-08
	SUPT16H	-17.9	1.20E-09		GUCY1B3	-13.3	7.41E-08
	GGA3	17.8	1.33E-09		#GLRB	-13.2	7.41E-08
	ZHX1	-17.5	1.61E-09		SMURF2	13.4	7.41E-08
	SCN2B	-17.1	2.35E-09		DZIP3	-13.4	7.41E-08
	OPA1	-17.0	2.49E-09		#CDC42	-13.2	7.41E-08
	SMS	-16.8	2.77E-09		EMC3	-13.3	7.41E-08
	CAMTA1	-16.5	3.81E-09		+PCSK1	-13.0	8.20E-08
	COPG1	-16.3	4.78E-09		VTA1	-12.9	8.20E-08

Supplementary Table 6. Top genes in IGN ranked by degree Top IGN genes with degree 6 or greater. Degree/rank of gene in xIGN given in 4th column. Symbols: #, gene common to three or more AD brain regions; +, gene common to two AD brain regions; *, gene is common to AG and at least one AD brain region; $, top-20 gene by degree in xIGN.

Dataset	Genes	Degree in IGN	Rank/degree in xIGN	Dataset	Genes	Degree in IGN	Rank/de-gree in xIGN
AG	PSMD4[$]	9	3/87	HC	RPL7	7	185/74
	PSMA3	7	143/20		PSMD8	7	54/147
	*PSMC2	7	24/52		CD19	6	798/6
	SSX2IP	6	136/21		[#]IGHG1	6	100/107
	PSMC5[$]	6	2/88		MDH1	6	31/166
	PSMD2[$]	6	6/78		PSMA6	6	72/130
	*PSMD6	6	50/42		PSMB7[$]	6	3/240
EC	[#]YWHAZ	11	109/85		*PSMC2	6	57/142
	PHF20	10	305/34		[+]PSMC6	6	33/165
	[#]TUBB	7	305/34		YWHAB	6	37/160
	[+]TUBA4A	7	113/83	MTG	[#]TUBB	14	75/143
	CLTC[$]	6	1/219		NSF[$]	10	4/252
	[#]IGHG1[$]	6	12/179		HSPA8	9	135/108
	PLA2G4F	6	111/84		[+]YWHAG	8	53/158
	[+]YWHAG	6	76/107		[#]YWHAZ[$]	8	18/201
	[+]ACTG1	6	113/83		[+]TUBA4A	8	44/167
	[+]STXBP1	6	61/116		TCP1	7	130/110
	[+]PSMC6[$]	6	6/188		PPP3CA	7	32/176
	CCT8	6	19/165		CDC42	6	102/127
HC	[#]YWHAZ	14	42/154		[+]STXBP1	6	235/62
	PSMA2	13	45/151		TIAM1	6	73/144
	ACTB	9	231/59		TRIM36	6	113/118
	*PSMD6[$]	9	17/178	PC	EEF2	6	74/61
	[#]TUBB	8	106/105		GAPDH	6	53/70
	CCT7[$]	7	4/237	SFG	[#]IGHG1	6	1/221
	RPL17	7	62/140				

Supplementary Table 7. Top-10 genes in xIGNs by degree Top-10 xIGN genes ranked by degree. Degree/rank of gene in IGN given in 4th column. Symbols: #, gene common to three or more AD brain regions; +, gene common to two AD brain regions; *, gene is common to AG and at least one of five AD brain regions; &, high-degree gene in IGN.

Dataset	Genes	Degree in xIGN	Rank/ degree in IGN	Dataset	Genes	Degree in xIGN	Rank/ degree in IGN
AG	*CCT7	90	22/2	MTG	MAP2K1	267	82/2
	PSMC5$^{\&}$	88	4/6		$^{+}$DYNC1I1	264	82/2
	PSMD4$^{\&}$	87	1/9		NAP1L5	255	148/1
	COPS5	83	64/1		NSF$^{\&}$	252	2/10
	VPS72	82	64/1		$^{+}$ATP6V1E1	249	52/3
	PSMD2$^{\&}$	78	4/6		RCAN2	238	148/1
	SUMO1	77	22/2		ATP6V1H	235	148/1
	RAF1	75	10/3		$^{+}$GABRG2	233	29/4
	IMMT	74	64/1		MAP4	224	82/2
	PSMC6	71	9/4		TRIM37	223	148/1
EC	CLTC$^{\&}$	219	5/6	PC	RNF11	138	3/5
	DCTN4	203	115/1		CAMK2B	120	24/2
	WASL	203	26/4		EEF1G	118	24/2
	UBE2N	196	64/2		PI4KA	113	66/1
	$^{+}$DYNC1I1	193	115/1		AP2M1	112	24/2
	PSMC6$^{\&}$	188	5/6		P4HB	112	66/1
	$^{+}$RAB6A	188	115/1		ATP2A2	111	66/1
	$^{+}$ATP6V1E1	186	37/3		CLTB	109	66/1
	HECTD1	185	115/1		RABAC1	108	66/1
	PSMC2	184	13/5		$^{+}$SCOC	108	24/2
HC	DDX1	270	73/2	SFG	IGHG1$^{\&}$	221	1/6
	NECAP1	254	46/3		EPS16	134	13/2
	PSMB7$^{\&}$	240	10/6		PRKX	117	44/1
	*CCT7$^{\&}$	237	6/7		USP32	109	44/1
	DSTN	226	46/3		ATP5A1	107	44/1
	CCT8	225	73/2		KPNB1	101	44/1
	$^{+}$RAB6A	211	134/1		SEC24C	97	44/1
	TOPBP1	205	134/1		H2AFZ	85	44/1
	$^{+}$SCOC	198	134/1		PRKY	84	44/1
	ATP5C1	195	33/4		$^{+}$GABRG2	77	4/3

Supplementary Table 8. Enrichment of known AD target genes in curated AG and ALZ gene sets Enrichments of top AlzGane and AlzBase gene sets are shown as number of hits and, in brackets, Fisher's exact test *p*-value. Sizes of curated gene sets are given in Supplementary Table 4. GOC, gain of co-expression; LOC, loss of co-expression. Complete list of known target genes enriched in curated gene sets may be accessed at the Figshare database link: 10.6084/m9.figshare.8952938

Gene sets	AG	EC	HC	MTG	PC	SFG
Enrichment in DEG sets						
Top-106 AlzGene target genes No. of hits (*p*-value)	7 (7.0E-02)	9 (2.2E-02)	3 (7.8E-01)	2 (9.2E-01)	5 (3.2E-01)	3 (7.7E-01)
Rank of highest ranking gene	49	160	358	40	23	674
Up-/down-regulated	4/3	3/6	0/3	1/1	1/4	1/2
Top-109 AlzBase target genes No. of hits (*p*-value)	63 (7.5E-64)	11 (3.4E-03)	23 (2.1E-11)	42 (2.3E-31)	28 (6.3E-17)	52 (1.9E-44)
Rank of highest ranking gene	3	92	51	52	2	2
Up-/down-regulated	11/52	6/5	0/23	11/31	0/28	6/46
Enrichment in IGN sets						
Top-106 AlzGene target genes No. of hits (*p*-value)	9 (1.9E-05)	3 (2.1E-01)	3 (3.0E-01)	3 (2.8E-01)	7 (1.3E-03)	7 (4.4E-04)
Rank of highest ranking gene	10	13	19	13	1	44
Genes with GOC/LOC/both links	7/4/2	1/2/0	1/3/1	2/2/1	3/4/0	3/4/0
Top-109 AlzBase target genes No. of hits (*p*-value)	9 (2.4E-05)	19 (3.0E-15)	19 (7.7E-14)	28 (4.6E-25)	12 (1.2E-07)	13 (1.5E-09)
Rank of highest ranking gene	10	26	11	2	24	2
Genes with GOC/LOC/both links	8/1/0	4/17/2	16/6/3	21/13/6	6/7/1	6/7/0
Enrichment in xIGN sets						
Top-106 AlzGene target genes No. of hits (*p*-value)	12 (1.5E-03)	5 (3.4E-01)	7 (2.6E-01)	8 (7.4E-02)	9 (3.2E-02)	10 (1.5E-03)
Rank of highest ranking gene	38	51	57	52	13	18
Genes with GOC/LOC/both links	10/10/8	5/4/4	6/7/6	7/7/6	8/9/8	10/9/9
Top-109 AlzBase target genes No. of hits (*p*-value)	17 (2.4E-06)	29 (1.4E-17)	29 (3.5E-14)	38 (2.9E-25)	25 (2.1E-12)	23 (1.9E-13)
Rank of highest ranking gene	60	5	2	2	16	10
Genes with GOC/LOC/both links	17/4/13	28/29/28	28/25/24	38/37/37	22/20/17	22/19/18

Supplementary Table 9. DCE partners of five culprit genes A DCE partner a culprit gene is included if the magnitude in the change in linear regression coefficient, |Δ*r*|, of the gene-pair is greater than 1.5, and if the pair passes the Q-statistic cutoff (see Supplementary Table 3). These are not necessarily IDCE partners, or genes in the appropriate IGN, and most are not. Culprit gene symbols stand for: *APP*, amyloid precursor protein; *MAPT*, tau protein; *APOE*, apolipoprotein E; *PSEN1*, presenilin-1; *PSEN2*, presenilin-2.

Dataset	Culprit gene	GOC and LOC partners
HC	APP	**GOC** – KDM2A (1.85), DDHD1 (1.80)
	MAPT	**GOC** – GABRA1 (1.87), CDH18 (1.84), GABRD (1.70)
	APOE	**GOC** – BAK1 (1.88)<br>**LOC** – PUM1 (-1.81), DR1 (-1.81)
	PSEN2	**GOC** – POLR2H (1.80), ZWILCH (1.64), C1orf115 (1.56), SORBS2 (1.52)<br>**LOC** – ZNF606 (-1.86), NRP2 (-1.65)
MTG	APP	**GOC** – PDE2A (1.57), OGDHL (1.52)<br>**LOC** – KRAS (-1.65), ZBTB39 (-1.52)
PC	MAPT	**LOC** – LDB3 (-1.75), ST6GALNAC1 (-1.62), AIP (-1.54)
SFG	APP	**GOC** – SETD5 (1.73), CDK5RAP3 (1.73), TYK2 (1.72), EIF2B4 (1.65), PRPF8 (1.50)<br>**LOC** – CLNK (-1.75), UCKL1 (-1.72), VENTXP1 (-1.67), BPIFA3 (-1.63), ARL2BP (-1.51)
	APOE	**GOC** – NFU1 (1.86), SEC61G (1.79), ASAH1 (1.75), G2E3 (1.73), SH3BGRL (1.72), PAPD4 (1.69), MIR22 (1.63), MIR22HG (1.63), TMEM177 (1.60), NDUFB6 (1.57), BHLHE40 (1.54), LACTB2 (1.54), TMEM14B (1.53), TMEM14C(1.53), SPAG9(1.51)<br>**LOC** – MROH6 (-1.76), INSC (-1.75), BTBD8 (-1.75), CYMP (-1.74), C1orf180 (-1.71), FLJ20518 (-1.70), C6orf118 (-1.56), ARMCX6 (-1.56), ADAMTS21 (-1.56)
	PSEN1	**LOC** – CEBPA (-1.63), RHOD (-1.55)
	MAPT	**GOC** – PCDH7 (1.80), BRI3BP (1.80), ZC3H15 (1.78), EPHA4 (1.76), FAM3C (1.76), AKAP11 (1.76), MAPK9 (1.75), ENOPH1 (1.75), CAMTA1 (1.73), H2AFZ (1.73), SEC62 (1.73), MYO5A (1.72), SLC16A14 (1.71), PPP3CA (1.71), MRPL15 (1.71), MGAT5 (1.71), SACS (1.67), BID (1.67), PNMAL1 (1.67), KLHL7 (1.64), USP32 (1.63), CDR2 (1.62), C1QTNF4 (1.60), KIAA1244 (1.60), ZFAND2A (1.60), TDP2 (1.58), ITCH (1.56), SMIM19 (1.55), ARHGAP32 (1.50)<br>**LOC** – HDAC6 (-1.76), LDLRAD4-AS1 (-1.72), LIMS2 (-1.72), NKX6-2 (-1.71), BCAR4 (-1.71), PIP4K2B (-1.70), ADRA1A (-1.70), HK3 (-1.70), SLC1A5 (-1.66), SUSD2 (-1.65), RASSF6 (-1.58), B2M (-1.55)

Supplementary Table 10. Culprit genes in curated AG and ALZ gene sets For IGN and xIGN number given is degree, and for DEG, *t*-score. Number in bracket is gene rank, by degree in IGN and xIGN, and by *t*-score in DEG. Up(down)-regulated DEGs have *t*-score >(<) 0. Culprit gene symbols stand for: *APP*, amyloid precursor protein; *MAPT*, tau protein; *APOE*, apolipoprotein E; *PSEN1*, presenilin-1; *PSEN2*, presenilin-2. Bars indicate genes do not appear in gene set.

IGN						
Culprit gene	AG (305 genes)	EC (325 genes)	HC (389 genes)	MTG (378 genes)	PC (335 genes)	SFG (278 genes)
APP	–	–	–	–	–	–
MAPT	1 (44)	–	–	–	–	–
APOE	2 (64)	–	–	–	–	1(44)
PSEN1	–	–	–	–	–	–
PSEN2	–	–	–	–	–	–
xIGN						
Culprit gene	AG (901 genes)	EC (787 genes)	HC (1063 genes)	MTG (903 genes)	PC (903 genes)	SFG (664 genes)
APP	–	–	–	14 (555)	–	10 (245)
MAPT	19 (149)	–	–	–	11 (478)	9 (270)
APOE	16 (175)	–	–	–	–	11 (213)
PSEN1	–	–	–	–	–	–
PSEN2	–	–	25 (422)	–	–	–
DEGs						
Culprit gene	AG (741 genes)	EC (845 genes)	HC (837 genes)	MTG (829 genes)	PC (764 genes)	SFG (829 genes)
APP	–	–	–	–	–	–
MAPT	–	–	–	–	–	–
APOE	–	–	–	–	–	–
PSEN1	–	–	–	–	-6.96 (612)	–
PSEN2	–	–	–	-8.20 (444)	–	–

Supplementary Table 11. KEGG enrichment in curated AG and ALZ gene sets Number of KEGG pathways enriched (DAVID v 6.7, Fisher's exact test p <0.05) in curated DEG, IGN, and xIGN gene sets; number of genes in each curated set is given in brackets.

Number of enriched KEGG pathways	AG	EC	HC	MTG	PC	SFG
DEG	30 (741 genes)	4 (845 genes)	18 (837 genes)	18 (829 genes)	10 (764 genes)	13 (829 genes)
IGN	34 (305 genes)	13 (325 genes)	18 (389 genes)	25 (378 genes)	25 (335 genes)	28 (278 genes)
xIGN	32 (901 genes)	18 (787 genes)	21 (1063 genes)	33 (903 genes)	44 (903 genes)	35 (664 genes)

Supplementary Table 12. Top-10 KEGG pathways enriched in DEG Top-10 KEGG pathways enriched in DEG sets (DAVID v 6.7, Fisher's exact test *p*<0.05). Asterisks mark KEGG pathways that are also top-10 in same-case IGN and/or xIGN sets.

Datasets	KEGG pathway	*p*-value	Datasets	KEGG pathway	*p*-value
AG	hsa04010:MAPK signaling pathway	7.38E-07	MTG	hsa05120:Epithelial cell signaling in Helicobacter pylori infection	1.19E-04
	hsa04514:Cell adhesion molecules (CAMs)	6.28E-06		hsa00190:Oxidative phosphorylation	2.41E-04
	hsa05120:Epithelial cell signaling in Helicobacter pylori infection	1.07E-04		hsa05110:Vibrio cholerae infection	4.00E-04
	hsa04020:Calcium signaling pathway	1.45E-04		hsa04520:Adherens junction	1.47E-03
	hsa04720:Long-term potentiation	4.56E-04		hsa05412:Arrhythmogenic right ventricular cardiomyopathy (ARVC)	4.42E-03
	hsa05020:Prion diseases	1.41E-03		hsa05130:Pathogenic Escherichia coli infection	7.15E-03
	hsa04730:Long-term depression	1.97E-03		hsa05211:Renal cell carcinoma	7.92E-03
	hsa04114:Oocyte meiosis	2.95E-03		hsa04330:Notch signaling pathway	8.43E-03
	hsa04070:Phosphatidylinositol signaling system	3.34E-03		hsa04810:Regulation of actin cytoskeleton	1.13E-02
	hsa05322:Systemic lupus erythematosus	3.43E-03		hsa04510:Focal adhesion	1.18E-02
EC	hsa00030:Pentose phosphate pathway	5.17E-04	PC	hsa05012:Parkinson's disease	2.18E-23
	hsa00051:Fructose and mannose metabolism	2.84E-03		hsa00190:Oxidative phosphorylation	3.82E-21
	hsa04666:Fc gamma R-mediated phagocytosis	7.08E-03		hsa05016:Huntington's disease	7.54E-18
	*hsa05016:Huntington's disease	2.16E-02		hsa05010:Alzheimer's disease	1.79E-15
	–	–		*hsa03050:Proteasome	1.94E-09
				*hsa05130:Pathogenic Escherichia coli infection	1.08E-03
				hsa00620:Pyruvate metabolism	6.09E-03
				hsa04260:Cardiac muscle contraction	1.11E-02
				*hsa03040:Spliceosome	2.33E-02
				hsa00020:Citrate cycle (TCA cycle)	2.73E-02
HC	*hsa05016:Huntington's disease	3.46E-13	SFG	hsa00190:Oxidative phosphorylation	6.46E-13
	*hsa05012:Parkinson's disease	7.77E-12		hsa05012:Parkinson's disease	1.17E-06
	*hsa00190:Oxidative phosphorylation	8.08E-09		hsa05016:Huntington's disease	2.71E-06
	*hsa03050:Proteasome	1.82E-08		hsa00020:Citrate cycle (TCA cycle)	1.37E-05
	*hsa05010:Alzheimer's disease	6.97E-08		hsa03050:Proteasome	1.47E-05
	hsa00620:Pyruvate metabolism	2.89E-04		hsa05120:Epithelial cell signaling in Helicobacter pylori infection	2.57E-05
	hsa00970:Aminoacyl-tRNA biosynthesis	3.53E-04		hsa05010:Alzheimer's disease	5.59E-05
	hsa00020:Citrate cycle (TCA cycle)	1.35E-03		hsa05110:Vibrio cholerae infection	4.00E-04
	hsa00290:Valine, leucine and isoleucine biosynthesis	2.36E-03		hsa00620:Pyruvate metabolism	3.37E-03
	hsa05110:Vibrio cholerae infection	3.63E-03		*hsa05130:Pathogenic Escherichia coli infection	7.15E-03

Supplementary Table 13. Top-10 KEGG pathways enriched in IGN Top-10 KEGG pathways enriched in IGN sets (DAVID v 6.7, Fisher's exact test P <0.05). Asterisks/#'s mark KEGG pathways that are also top-10 in same-case DEG/xIGN sets.

Data sets	KEGG pathway	*p*-value	Data sets	KEGG pathway	*p*-value
AG	#hsa03050:Proteasome	2.44E-16	MTG	#hsa03050:Proteasome	8.47E-09
	#hsa05200:Pathways in cancer	9.26E-06		hsa05130:Pathogenic Escherichia coli infection	6.08E-06
	hsa05213:Endometrial cancer	1.75E-05		#hsa04666:Fc gamma R-mediated phagocytosis	4.33E-05
	*hsa05010:Alzheimer's disease	2.13E-05		#hsa03010:Ribosome	7.93E-05
	#hsa05130:Pathogenic Escherichia coli infection	3.76E-05		#hsa04144:Endocytosis	4.52E-04
	hsa05215:Prostate cancer	5.47E-05		#hsa04114:Oocyte meiosis	7.41E-04
	#hsa05220:Chronic myeloid leukemia	6.25E-05		hsa04012:ErbB signaling pathway	1.37E-03
	hsa05210:Colorectal cancer	1.66E-04		hsa04660:T cell receptor signaling pathway	2.15E-03
	#hsa04012:ErbB signaling pathway	2.22E-04		hsa04722:Neurotrophin signaling pathway	6.28E-03
	*#hsa05016:Huntington's disease	2.54E-04		hsa04110:Cell cycle	6.67E-03
EC	#hsa03050:Proteasome	3.33E-11	PC	*#hsa03050:Proteasome	3.04E-08
	#hsa04114:Oocyte meiosis	8.68E-05		#hsa03040:Spliceosome	9.13E-07
	hsa03010:Ribosome	2.22E-04		#hsa03010:Ribosome	1.02E-06
	#hsa04120:Ubiquitin mediated proteolysis	6.88E-04		#hsa05215:Prostate cancer	7.67E-06
	#hsa05130:Pathogenic Escherichia coli infection	1.34E-03		#hsa04144:Endocytosis	4.35E-05
	#hsa04540:Gap junction	4.63E-03		#hsa04110:Cell cycle	7.44E-05
	hsa04730:Long-term depression	1.60E-02		hsa05214:Glioma	2.41E-04
	*#hsa05016:Huntington's disease	1.74E-02		hsa05200:Pathways in cancer	1.53E-03
	hsa05014:Amyotrophic lateral sclerosis (ALS)	1.97E-02		*hsa05130:Pathogenic Escherichia coli infection	2.93E-03
	#hsa04110:Cell cycle	3.16E-02		hsa04520:Adherens junction	4.33E-03
HC	*#hsa03050:Proteasome	2.01E-18	SFG	#hsa04114:Oocyte meiosis	8.04E-06
	#hsa03010:Ribosome	1.42E-08		hsa04660:T cell receptor signaling pathway	3.43E-05
	*hsa05016:Huntington's disease	3.65E-05		#hsa05200:Pathways in cancer	7.34E-05
	hsa04114:Oocyte meiosis	5.31E-05		hsa03010:Ribosome	1.19E-04
	#hsa05130:Pathogenic Escherichia coli infection	2.32E-04		#hsa04722:Neurotrophin signaling pathway	5.49E-04
	*hsa05012:Parkinson's disease	2.77E-04		hsa04110:Cell cycle	5.88E-04
	*#hsa00190:Oxidative phosphorylation	1.08E-03		#hsa04360:Axon guidance	7.68E-04
	hsa04722:Neurotrophin signaling pathway	2.22E-03		*hsa05130:Pathogenic Escherichia coli infection	8.63E-04
	#hsa00010:Glycolysis / Gluconeogenesis	6.76E-03		hsa04662:B cell receptor signaling pathway	9.61E-04
	*hsa05010:Alzheimer's disease	7.86E-03		hsa04510:Focal adhesion	1.15E-03

Supplementary Table 14. Top-10 KEGG enriched in xIGN Top-10 KEGG pathways enriched in xIGN sets (DAVID v 6.7, Fisher's exact test P <0.05). Asterisks/#'s mark KEGG pathways that are also top-10 in same-case DEG/IGN sets.

Data sets	KEGG pathway	*p*-value	Data sets	KEGG pathway	*p*-value
AG	#hsa03050:Proteasome	6.39E-15	MTG	#hsa03050:Proteasome	6.88E-13
	hsa03040:Spliceosome	7.71E-09		hsa04120:Ubiquitin mediated proteolysis	1.77E-06
	#hsa05220:Chronic myeloid leukemia	3.08E-06		#hsa04144:Endocytosis	4.51E-06
	*#hsa05016:Huntington's disease	5.44E-05		*hsa05010:Alzheimer's disease	5.51E-05
	hsa03010:Ribosome	1.00E-04		#hsa03010:Ribosome	1.09E-04
	*hsa05010:Alzheimer's disease	1.73E-04		*hsa05016:Huntington's disease	1.34E-04
	#hsa05200:Pathways in cancer	2.00E-04		hsa05014:Amyotrophic lateral sclerosis (ALS)	1.66E-04
	#hsa05130:Pathogenic Escherichia coli infection	5.19E-04		#hsa04114:Oocyte meiosis	2.96E-04
	hsa05014:Amyotrophic lateral sclerosis (ALS)	1.00E-03		hsa05012:Parkinson's disease	3.44E-04
	#hsa04012:ErbB signaling pathway	0.00103		#hsa04666:Fc gamma R-mediated phagocytosis	3.50E-04
EC	#hsa03050:Proteasome	8.15E-18	PC	*#hsa03050:Proteasome	1.47E-09
	#hsa04120:Ubiquitin mediated proteolysis	1.11E-05		#hsa03040:Spliceosome	8.26E-07
	#hsa04114:Oocyte meiosis	3.50E-05		hsa04120:Ubiquitin mediated proteolysis	1.32E-06
	#hsa04110:Cell cycle	2.23E-04		#hsa03010:Ribosome	2.76E-06
	#hsa04540:Gap junction	2.44E-04		hsa04012:ErbB signaling pathway	2.76E-06
	hsa03040:Spliceosome	2.49E-04		hsa04910:Insulin signaling pathway	1.05E-05
	#hsa05130:Pathogenic Escherichia coli infection	3.25E-04		#hsa04144:Endocytosis	1.95E-05
	hsa04144:Endocytosis	5.30E-04		#hsa05215:Prostate cancer	5.58E-05
	*#hsa05016:Huntington's disease	8.98E-04		#hsa04110:Cell cycle	8.05E-05
	hsa04722:Neurotrophin signaling pathway	1.48E-03		hsa04914:Progesterone-mediated oocyte maturation	1.21E-04
HC	*#hsa03050:Proteasome	2.80E-15	SFG	hsa04666:Fc gamma R-mediated phagocytosis	1.67E-04
	*#hsa05016:Huntington's disease	2.73E-09		#hsa04360:Axon guidance	1.94E-04
	*#hsa05012:Parkinson's disease	5.79E-08		#hsa04722:Neurotrophin signaling pathway	3.62E-04
	#hsa03010:Ribosome	5.92E-07		hsa05220:Chronic myeloid leukemia	6.62E-04
	hsa04120:Ubiquitin mediated proteolysis	3.16E-06		hsa04012:ErbB signaling pathway	7.82E-04
	*#hsa00190:Oxidative phosphorylation	1.01E-05		#hsa05200:Pathways in cancer	8.29E-04
	#hsa00010:Glycolysis / Gluconeogenesis	1.12E-04		#hsa04114:Oocyte meiosis	8.47E-04
	hsa03040:Spliceosome	1.36E-04		hsa05215:Prostate cancer	9.74E-04
	#hsa05130:Pathogenic Escherichia coli infection	2.32E-04		hsa04540:Gap junction	9.74E-04
	*#hsa05010:Alzheimer's disease	2.47E-04		hsa04062:Chemokine signaling pathway	1.18E-03

Supplementary Table 15.1. Hits of KEGG pathway Pathogen *E coli*. infection in six cases

Gene symbol	DEG (*t*-score, rank)					
	AG (741 genes)	EC (845 genes)	HC (837 genes)	MTG (829 genes)	PC (764 genes)	SFG (829 genes)
ACTB	-	-	-8.71, 119	-13.82, 10	-	-
ACTG1	-	-	-	-	-	-
ARPC3	-	-	-	-	-6.92, 633	-
ARPC5L	-	-	-9.37, 356	-	-	-
ARPC1A	-	-	-12.55, 53	-	-6.82, 673	-7.54, 767
ARHGEF2	-	-	-	-	7.76, 339	-
CD14	5.34, 721	-	-	-	-	-
CDC42	-10.19, 98	-	-12.74, 44	-	-12.47, 2	-13.19, 3
CDH1	-	-	-	-	-	-
CTTN	-	-	-	7.30, 803	-	-
EZR	5.31, 725	-	-	11.14, 53	-	9.61, 174
FYN	-	-	-	-	-	-
HCLS1	6.77, 486	-	-	-	-	-
KRT18	-	-	-	-	-	-
LY96	7.12, 424	-	-	-	-	-
ITGB1	5.78, 641	-	-	9.71, 162	-	-
NCK1	-	-	-	-	-	-
NCL	-	-	-	-	-	-
TUBA1B	-	-	-9.16, 418	-8.03, 500	-7.53, 405	-7.54, 769
TUBA1C	-	-	-9.08, 436	-8.05, 487	-7.81, 324	-
TUBA3C	-	-	-	-10.09, 126	-7.49, 411	-
TUBA4A	-6.69, 497	-	-	-	-	-9.40, 205
TUBB	-	-	-12.66, 77	-	-7.24, 491	-9.91, 136
TUBB3	-	-	-	-8.67, 308	-6.94, 621	-7.71, 679
TUBB6	5.96, 607					
RHOA	-				-6.93, 627	
ROCK2	-	-	-	-	-	-
WAS	-	-	-	-	-	-
WASL	-	-	-	-	-	-9.58, 176
YWHAQ	-	-	-	-	-	-
YWHAZ	-13.10, 30	-	-8.48, 656	-7.58, 673	-8.52, 181	-7.54, 769
Fisher's exact test *p*-value	6.67E-03	-	3.92E-02	7.15E-03	1.08E-03	7.15E-03

Supplementary Table 15.2. Hits of KEGG pathway OXPHOS in six cases

Gene symbol	DEG (*t*-score, rank)					
	AG (741 genes)	EC (845 genes)	HC (837 genes)	MTG (829 genes)	PC (764 genes)	SFG (829 genes)
ATP5A1	-	-	-8.71, 562	-	-10.06, 49	-
ATP5B	-	-	-9.64, 303	-	-12.47, 2	-7.83, 632
ATP5C1	-	-	-	-8.77, 279	-10.20, 42	-7.91, 592
ATP5F1	-	-	-	-	-6.81, 678	-
ATP5G1	-	-10.18, 294	-	-9.94, 138	-	-10.70, 71
ATP5G3	-	-	-	-	-9.51, 86	-8.30, 454
ATP5H	-	-	-	-	-7.78, 331	-
ATP5J	-	-	-	-	-9.40, 93	-
ATP5J2	-	-7.89, 837	-	-7.95, 529	-6.73, 724	-7.57, 754
ATP5L	-	-	-	-7.64, 645	-7.96, 282	-7.90, 593
ATP5O	-	-	-10.29, 210	-	-10.02, 56	-7.98, 554
ATP6AP1	-	-	-	-	-	-8.57, 373
ATP6V0A1	-7.46, 382	-	-	-	-	-
ATP6V0B	-	-8.03, 783	-	-	-	-8.66, 351
ATP6V0C	-	-	-	-	-	-8.42, 413
ATP6V0D1	-6.85, 469	-	-	-	-	-7.98, 557
ATP6V0E1	8.39, 256	-	-	10.52, 84	-	-
ATP6V1A	-13.50, 21	-	-	-	-	-8.57, 371
ATP6V1B2	-9.30, 163	-	-9.13, 424	-8.30, 424	-7.43, 429	-11.43, 40
ATP6V1C1	-13.39, 22	-	-8.80, 529	-	-6.90, 644	-7.51, 784
ATP6V1D	-	-	-	-7.29, 814	-	-
ATP6V1E1	-	-	-11.26, 120	-9.01, 247	-6.63, 761	-11.36, 44
ATP6V1F	-	-	-	-8.51, 364	-	-8.75, 334
ATP6V1G2	-7.37, 395	-	-	-9.66, 167	-	-12.28, 22
ATP6V1H	-	-	-8.46, 666	-	-7.50, 411	-
COX3	-	-	-	8.63, 324	-	-
COX4I1	-	-	-8.88, 499	-	-7.45, 423	-
COX5A	-7.43, 387	-	-	-	-7.16, 536	-
COX5B	-	-	-9.83, 275	-	-9.21, 105	-
COX6A1	-	-	-	-	-6.81, 679	-
COX6B1	-	-	-9.81, 278	-11.4, 45	-7.13, 545	-
COX6C	-	-	-8.69, 574	-	-8.16, 272	-
COX7A2L	-	-	-	-	-	-9.06, 270
ND2	-	-	-	-	-	11.01, 60
ND6	-	-	-	-	7.12, 549	-
NDUFA1	-	-	-	-	-7.99, 272	-
NDUFA10	-	-	-12.36, 59	-	-	-
NDUFA11	-	-	-	-	-	-7.78, 653
NDUFA13	-	-	-10.39, 196	-	-	-
NDUFA2	-	-	-	-	-8.85, 136	-7.46, 819
NDUFA3	-	-9.48, 422	-	-	-	-
NDUFA4	-	-	-	-	-6.73, 721	-
NDUFA5	-	-	-	-	-10.60, 28	-

NDUFA6	-	-	-9.28, 387	-	-	-
NDUFA8	-	-	-9.21, 403	-8.00, 516	-	-8.14, 503
NDUFA9	-	-	-8.96, 469	-	-7.51, 409	-9.77, 162
NDUFAB1	-	-	-8.69, 575	-8.39, 393	-6.97, 600	-11.13, 54
NDUFB10	-	-	-10.41, 192	-	-8.22, 226	-
NDUFB11	-	-	-	-9.09, 239	-	-
NDUFB4	-	-8.66, 612	-	-	-7.09, 560	-
NDUFB5	-	-	-	-	-7.82, 323	-
NDUFB6	-	-	-	-	-7.67, 366	-
NDUFB8	-	-	-9.11, 427	-7.37, 778	-	-
NDUFB9	-	-	-	-	-7.33, 463	-
NDUFC2	-	-	-	-	-9.66, 76	-9.00, 279
NDUFS2						
NDUFS3	-	-	-10.55, 172	-	-7.98, 280	-10.19, 110
NDUFS4	-	-	-	-	-6.67, 744	-
NDUFS5	-	-	-10.68, 161	-7.34, 786	-8.12, 245	-9.24, 234
NDUFS6	-	-	-8.43, 674	-	-	-
NDUFS7	-	-8.73, 600	-8.94, 475	-	-	-
NDUFV2	-	-	-9.06, 438	-	-6.95, 617	-8.15, 502
PPA1	-	-	-	-	-8.03, 264	-
PPA2	-	-	-	7.49, 703	-	-
SDHA	-	-	-10.81, 151	-	-8.57, 172	-
SDHB	-	-	-	-	-9.07, 115	-
UQCR10	-	-	-9.28, 386	-	-	-7.60, 748
UQCRC1						
UQCRC2	-	-	-10.19, 220	-	-14.35, 1	-9.12, 254
UQCRFS1	-9.12, 179	-	-	-	-8.81, 140	-
UQCRH	-	-	-8.65, 591	-9.60, 171	-	-9.68, 169
UQCRQ						
Fisher's exact test *p*-value	-	-	8.08E-09	2.41E-04	3.82E-21	6.46E-13

Supplementary Table 15.3. Hits of KEGG Proteasome in six cases

Gene symbol	DEG (*t*-score, rank)					
	AG (741 genes)	EC (845 genes)	HC (837 genes)	MTG (829 genes)	PC (764 genes)	SFG (829 genes)
POMP	–	–	-9.18, 410	–	-6.74, 719	–
PSMA1	–	–	-8.18, 768	-8.63, 328	-8.91, 127	-12.36, 16
PSMA2	–	–	–	–	–	-7.67, 708
PSMA3	–	–	–	–	-9.46, 90	–
PSMA4	–	–	–	–	–	–
PSMA5	-5.31, 726				-12.14, 7	-9.18, 242
PSMA6	–	–	–	–	–	–
PSMA7	–	–	–	-7.39, 764	–	-7.92, 583
PSMB1	–	–	-10.54, 176	–	-9.68, 76	–
PSMB2	–	–	-10.01, 244	–	–	-8.93, 293

PSMB3	–	–	-9.96, 253	–	-7.93, 295	-7.69, 693
PSMB4	–	–	-8.85, 510	–	-11.57, 11	–
PSMB5	–	–	–	–	-8.67, 156	-7.71, 680
PSMB6	–	–	-9.82, 276	–	-8.65, 160	–
PSMB7	–	–	–	–	-10.99, 15	–
PSMC1	–	–	-8.52, 639	–	-9.24, 102	-8.42, 414
PSMC2	–	–	-8.55, 619	–	-7.85, 312	-9.33, 213
PSMC3	–	–	–	–	–	–
PSMC4	–	–	–	–	–	–
PSMC5	–	–	-8.28, 725	-8.12, 465	-7.70, 357	–
PSMC6	–	–	–	–	–	–
PSMD1	–	–	–	-9.28, 219	–	-8.63, 357
PSMD2	–	–	–	–	–	-7.55, 765
PSMD4	–	–	-9.86, 271	–	-8.34, 206	–
PSMD6	–	–	–	–	-7.87, 307	–
PSMD7	-7.75, 433	–	-8.42, 676	–	–	–
PSMD8	–	–	–	–	-7.82, 322	-9.16, 247
PSMD11	–	–	-8.71, 567	–	–	–
PSMD12	–	–	-13.21, 34	–	-7.54, 400	–
PSMD13	–	–	-8.28, 728	-10.55, 82		
PSMD14	–	–	-10.19, 220	–		
Fisher’s exact test *p*-value	–	–	1.82E-08	–	1.94E-09	1.47E-05

Supplementary Table 15.4. Hits of KEGG pathway *Vibrio cholerae* infection in six cases

Gene symbol	DEG (*t*-score, rank)					
	AG (741 genes)	EC (845 genes)	HC (837 genes)	MTG (829 genes)	PC (764 genes)	SFG (829 genes)
ACTB	-	-	-11.28, 119	-13.82, 10	-	-
ARF1	-	-	-9.51, 332	-	-	-
ATP6AP1	-	-	-	-	-	-8.57, 373
ATP6V0A1	-7.46, 382	-	-	-	-	-
ATP6V0B	-	-8.03, 783	-	-	-	-8.66, 351
ATP6V0C	-	-	-	-	-	-8.42, 413
ATP6V0D1	-6.85, 469	-	-	-	-	-7.98, 557
ATP6V0E1	8.39, 256	-	-	10.52, 84	-	-
ATP6V1A	-13.51, 21	-	-	-	-	-8.57, 371
ATP6V1B2	-9.30, 163	-	-9.13, 424	-8.30, 424	-7.43, 429	-11.43, 40
ATP6V1C1	-13.39, 22	-	-8.80, 529	-	-6.90, 644	-7.51, 784
ATP6V1D	-	-	-	-7.29, 814	-	-
ATP6V1E1	-	-	-11.26, 120	-9.01, 247	-6.63, 761	-11.36, 44
ATP6V1F	-	-	-	-8.51, 364	-	-8.75, 334
ATP6V1G2	-7.37, 395	-	-	-9.66, 167	-	-12.28, 22
ATP6V1H	-	-	-8.46, 666	-	-7.50, 411	-

GNAS	-	-	-9.36, 358	-9.63, 169	-	-
KDELR1	-	9.70, 379	10.37, 196	7.57, 675	-	-
KDELR2	-	-	-8.36, 696	-	-	-
PRKACB	-9.76, 128	-	-	-	-	-7.47, 810
PRKCB	-15.04, 7	-	-	-	-	-
SEC61G	-	-	-8.55, 619	-	-	-
TJP1	-	-	-	8.48, 369	-	-
TJP2	-	9.22, 484	-	10.05, 131	-	-
Fisher's exact test *p*-value	5.99E-03	-	3.63E-03	4.00E-04	-	4.00E-04

Supplementary Table 15.5. Hits of KEGG Pyruvate metabolism in six cases

Gene symbol	DEG (*t*-score, rank)					
	AG (741 genes)	EC (845 genes)	HC (837 genes)	MTG (829 genes)	PC (764 genes)	SFG (829 genes)
ACACA	-		-8.97, 465	-	-	-
ACACB	-		-	10.58, 78	-	9.37, 206
ACAT1	-		-8.98, 463	-	-	-
ACYP2	-		-9.30, 381	-	-8.10, 254	-
AKR1B1	-		-8.17, 770	-	-	-
DLD	-		-9.68, 296	-	-9.16, 111	-7.67, 706
GLO1	-		-8.27, 733	-7.58, 674	-10.09, 49	-
GRHPR	-		-8.96, 467	-	-	-
HAGH	-		-	-	-8.88, 131	-
LDHA	-		-	-	-	-10.06, 118
LDHB	-		-10.53, 178	-	-9.08, 115	-
MDH1	-9.66, 135		-	-9.33, 206	-8.31, 211	-8.44, 407
MDH2	-		-8.85, 510	-	-	-11.58, 36
ME1	-		-	-	-6.85, 660	-
ME3	-		-	-8.05, 489	-	-7.46, 819
PDHA1	-		-8.53, 632	-	-	-8.16, 497
PDHB	-		-	-	-7.21, 508	-7.76, 657
Fisher's exact test *p*-value	-	-	2.89E-04	-	6.09E-03	3.37E-03

Supplementary Table 15.6. Hits of KEGG TCA cycle in six cases

Gene symbol	DEG (*t*-score, rank)					
	AG (741 genes)	EC (845 genes)	HC (837 genes)	MTG (829 genes)	PC (764 genes)	SFG (829 genes)
ACLY	-	-	-8.36, 696	-	-	-
ACO2	-	-	-9.57, 319	-	-	-
DLD	-	-	-9.68, 296	-	-9.16, 101	-7.67, 706
FH	-	-	-	-7.41, 749	-7.21, 508	-9.78, 156

IDH3A	-8.50, 242	-	-12.07, 80	-	-	-8.36, 437
IDH3B	-	-	-	-	-	-7.46, 825
IDH3G	-	-9.71, 378	-8.04, 834	-	-	-8.62, 358
MDH1	-9.66, 135	-	-	-9.33, 206	-8.31, 211	-8.44, 407
MDH2	-	-	-8.85, 510	-	-	-11.58, 36
PDHA1	-	-	-8.53, 632	-	-	-8.16, 497
PDHB	-	-	-	-	-7.21, 508	-7.76, 657
SDHA	-	-	-10.81, 151	-	-8.57, 172	-
SUCLA2	-7.14, 420	-	-	-	-9.07, 115	-8.98, 283
Fisher's exact test *p*-value	-	-	1.35E-03	-	2.73E-02	1.37E-05

Supplementary Table 16. Fifty-four "ALZ-only" hits in leading enriched KEGG pathways *t*-scores (gene rank by FDR in bracket) of hits from leading KEGG pathways enriched in DEG sets of ALZ, but not of AG. Up(down)-regulated DEGs have *t*-score >(<) 0.

"ALZ-only" hits						
Gene symbols	AG (741 genes)	EC (845 genes)	HC (837 genes)	MTG (829 genes)	PC (764 genes)	SFG (829 genes)
ACYP2	-	-	-9.30 (381)	-	-8.10 (254)	-
ARPC1A	-	-	-12.55 (53)	-	-6.82 (673)	-7.54 (767)
ATP5A1	-	-	-8.71 (562)	-	-10.06 (49)	-
ATP5B	-	-	-9.64 (303)	-	-12.47 (2)	-7.83 (632)
ATP5C1	-	-	-	-8.77 (279)	-10.20 (42)	-7.91 (592)
ATP5G3	-	-	-	-	-9.51 (86)	-8.30 (454)
ATP5J2	-	-7.89 (837)	-	-7.95 (529)	-6.73 (724)	-7.57 (754)
ATP5L	-	-	-	-7.64 (645)	-7.96 (282)	-7.90 (593)
ATP5O	-	-	-10.29 (210)	-	-10.02 (56)	-7.98 (554)
ATP6V1E1	-	-	-11.26 (120)	-9.01 (247)	-6.63 (761)	-11.36 (44)
ATP6V1H	-	-	-8.46 (666)	-	-7.50 (411)	-
COX4I1	-	-	-8.88 (499)	-	-7.45 (423)	-
COX5B	-	-	-9.83 (275)	-	-9.21 (105)	-
COX6B1	-	-	-9.81 (278)	-11.42 (45)	-7.13 (545)	-
COX6C	-	-	-8.69 (574)	-	-8.16 (238)	-
DLD	-	-	-9.68 (296)	-	-9.16 (111)	-7.67 (706)
FH	-	-	-	-7.41 (749)	-7.21 (508)	-9.78 (156)
GLO1	-	-	-8.27 (733)	-7.58 (674)	-10.09 (49)	-
IDH3G	-	-9.71 (378)	-8.04 (834)	-	-	-8.62 (358)
LDHB	-	-	-10.53 (178)	-	-9.08 (115)	-
MDH2	-	-	-8.85 (510)	-	-	-11.58 (36)
NDUFA2	-	-	-	-	-8.85 (136)	-7.46 (819)
NDUFA8	-	-	-9.21 (403)	-8.00 (516)	-	-8.14 (503)
NDUFA9	-	-	-8.96 (469)	-	-7.51 (409)	-9.77 (162)
NDUFAB1	-	-	-8.69 (575)	-8.39 (393)	-6.97 (600)	-11.13 (54)
NDUFB10	-	-	-10.41 (192)	-	-8.22 (226)	-
NDUFC2	-	-	-	-	-9.66 (76)	-9.00 (279)
NDUFS3	-	-	-10.55 (172)	-	-7.98 (280)	-10.19 (110)
NDUFS5	-	-	-10.68 (161)	-7.34 (786)	-8.12 (245)	-9.24 (234)
NDUFV2	-	-	-9.06 (438)	-	-6.95 (617)	-8.15 (502)
PDHA1	-	-	-8.53 (632)	-	-	-8.16 (497)
PDHB	-	-	-	-	-7.21 (508)	-7.76 (657)
POMP	-	-	-9.18 (410)	-	-6.74 (719)	-
PSMA1	-	-	-8.18 (768)	-8.63 (328)	-8.91(127)	-12.36 (16)
PSMB1	-	-	-10.54 (176)	-	-9.68 (76)	-

PSMB2	-	-	-10.01 (244)	-	-	-8.93 (293)
PSMB3	-	-	-9.96 (253)	-	-7.93 (295)	-7.69 (693)
PSMB4	-	-	-8.85 (510)	-	-11.57 (11)	-
PSMB5	-	-	-	-	-8.67 (156)	-7.71 (680)
PSMB6	-	-	-9.82 (276)	-	-8.65 (160)	-
PSMC1	-	-	-8.52 (639)	-8.12 (465)	-9.24 (102)	-8.42 (414)
PSMC2	-	-	-8.55 (619)	-	-7.85 (312)	-9.33 (213)
PSMC5	-	-	-8.28 (725)	-	-7.70 (357)	-
PSMD12	-	-	-13.21 (34)	-	-7.54 (400)	-7.55 (765)
PSMD4	-	-	-9.86 (271)	-	-8.34 (206)	-
PSMD8	-	-	-	-	-7.82 (322)	-9.16 (247)
SDHA	-	-	-10.81 (151)	-	-8.57 (172)	-
TUBA1B	-	-	-9.16 (418)	-8.03 (500)	-7.53 (405)	-7.54 (769)
TUBA1C	-	-	-9.08 (436)	-8.05 (487)	-7.81 (324)	-
TUBB	-	-	-12.66 (47)	-	-7.24 (491)	-9.91 (136)
TUBB3	-	-	-	-8.67 (308)	-6.94 (621)	-7.71 (679)
UQCR10	-	-	-9.28 (386)	-	-	-7.60 (748)
UQCRC2	-	-	-10.19 (220)	-	-14.35 (1)	-9.12 (254)
UQCRH	-	-	-8.65 (591)	-9.60 (171)	-	-9.68 (169)

Supplementary Table 17. Metrics of *REST* gene of AG and five ALZ datasets Gene is not a DEG in any of the datasets and is up-regulated in all cases.

	Dataset	*t-score*	*p*-value	FDR	FC	DEG (up/down)	FDR thresholds in our study
REST	AG	4.23	3.66E-04	4.15E-03	0.695	UP	7.50E-04
	EC	4.39	2.43E-04	1.72E-03	0.545	UP	4.00E-06
	HC	5.00	9.01E-05	7.97E-04	0.756	UP	1.00E-05
	MTG	4.41	1.66E-04	1.22E-03	0.689	UP	5.00E-06
	PC	2.34	3.00E-02	7.23E-02	0.541	UP	1.00E-04
	SFG	4.96	6.68E-05	7.19E-04	0.914	UP	1.25E-05

Supplementary Table 18. Comparison of DEG selection with authors of ALZ dataset
Comparison of DEG selection in current study and in (Liang et al., 2008a) (case A), where the selection criteria were (Bonferroni & Hochberg) FDR > 1.00E-02 and (approximately) |FC (log2)| > 0.58. In cases B and C, FDR threshold was determined by DEG set size. *Only 395 genes selected from SFG in case B. Original and reduced datasets described in Supplementary Table 1.

Dataset	A Original set From Supp. Mat. in (Liang et al., 2008a)		Analyzed by LIMMA			Overlap		
			|FC|>1, FDR determined by DEG set size	B Original set	C Reduced set (this study)			
	Probes	Genes	DEG set size	FDR threshold	FDR threshold	A&B	A&C	B&C
EC	4,025	3,091	845	2.28E-04	4.00E-06	777	751	609
HC	5,308	3,812	837	4.56E-03	1.00E-05	798	790	336
MTG	7,650	5,443	829	1.39E-04	5.00E-06	801	805	416
PC	4,886	3,592	764	1.27E-03	1.00E-04	691	717	359
SFG	1,176	972	*829	*1.00E-02	1.25E-05	92	245	92

Supplementary Table 19. Mitochondrial genes in OXPHOS, AD, PD, and HD pathways and in AG and ALZ DEG gene sets Top box, number of six families of mitochondrial genes and other genes in the 10 gene sets. Bottom box, Distribution of pathway specific genes in the 10 gene sets. Abbreviation: ND, NADH dehydrogenase; NDU, NADH: ubiquinone oxidoreductase; SDH, succinate dehydrogenase complex; COX, cytochrome c oxidase; ATP, ATPase; UQCR, ubiquinol-cytochrome c reductase; OXP, OXPHOS; AD Alzheimer's disease; PD, Parkinson's disease; HD, Huntington's disease. For complete list of genes see: Figshare database link at 10.6084/m9.figshare.8952938

Gene		KEGG gene sets				AG and ALZ DEG sets					
		OXP (133)	AD (171)	PD (142)	HD (193)	AG (741)	EG (845)	HC (837)	MTG (829)	PC (764)	SFG (829)
Mitochondrial	ND	7	0	7	0	0	0	0	0	1	1
	NDU	40	40	40	40	0	5	13	5	17	10
	SDH	4	4	4	4	0	0	1	0	2	0
	COX	24	20	20	20	1	0	4	2	6	1
	ATP	43	14	13	14	7	4	7	10	13	17
	UQCR	9	9	9	9	1	0	3	1	2	3
Others		6	2	2	2	0	0	0	1	1	0
Common with OXP		133	89	95	89	9	9	28	19	42	32
AD specific		0	68	0	0	8	2	7	6	6	5
PD specific		0	0	34	0	3	2	3	2	4	1
PD&HD specific		0	0	8	8	2	0	5	1	5	3
HD specific		0	0	0	83	1	7	11	6	5	6

Supplementary Table 20. The 32 novel AD genes
By definition the DEG set and the gene list of KEGG AD pathway contain no novel AD genes.

No. of 32 novel AD genes in curated gene sets						
dataset	AG	EC	HC	MTG	PC	SFG
DEG	0	2	25	10	28	21
IGN	10	12	17	13	9	6
xIGN	14	22	22	18	15	9
Enrichment Fisher exact *p*-value of original AD pathway						
dataset	AG	EC	HC	MTG	PC	SFG
DEG	-	-	6.97E-08	3.42E-02	1.79E-15	5.59E-05
IGN	2.13E-05	-	7.87E-03	1.82E-02	5.01E-02	7.94E-02
xIGN	1.73E-04	-	2.47E-04	5.51E-05	-	3.20E-02
Enrichment Fisher exact *p*-value of AD pathway augmented with novel AD genes						
dataset	AG	EC	HC	MTG	PC	SFG
DEG	-	-	1.90E-24	1.24E-05	3.38E-38	2.50E-17
IGN	9.86E-12	4.94E-08	3.42E-12	1.01E-08	8.52E-06	2.86E-04
xIGN	4.31E-10	8.86E-11	9.22E-14	9.60E-13	3.40E-06	5.10E-05
